# Epigenetic engineering and the art of epigenetic manipulation

**DOI:** 10.1186/gb4179

**Published:** 2014-06-24

**Authors:** Luca Magnani

**Affiliations:** 1 Division of Cancer Imperial Centre for Translational and Experimental Medicine, Imperial College London, London W12 0NN, UK

## Abstract

A report on the Epigenetic Engineering Meeting hosted by the Barts Institute of Cancer, held in London, UK, May 7, 2014

## 

This short and focused meeting was set up to bring the audience up to date with a specific set of techniques broadly categorized as epigenetic engineering (or epigenetic editing). The speakers reminded us of very important questions that still linger, like Damocles’ sword, over the field of epigenetics: what is the real role of epigenetics during gene transcription and development? Are epigenetic modifications the cause or consequence of these processes? While the jury is still out, this meeting reminded us that the tools needed to answer these philosophical questions are finally available and improving by the hour. Moreover, there was also a general consensus that epigenetic editing might provide the next big advance in cancer treatment. Epigenetic-editing treatments might finally allow targeted modulation of gene expression and could bypass the side effects of current epigenetic drugs.

## Two thumbs down to epigenetics haters?

Epigenetics has been defined in several ways, but none of these definitions includes an answer to the recurrent question: what are the actual roles of epigenetic modifications? With the explosion of ChIP-seq-based studies, we have now gathered epigenetic information (DNA methylation and histone modification maps) for hundreds of cell lines. We know that there is a strong correlation between certain epigenetic marks and the transcriptional status of nearby genes. We also understand that epigenetic information represents a sort of cellular barcode that is constantly modified during differentiation, and probably throughout cancer initiation and progression. Nevertheless, these ChIP-seq studies are frequently labeled as descriptive and correlative, and as not offering any answers to the consequentiality or temporal hierarchy between epigenetic and transcriptional reprogramming.

Tomasz Jurkowski (University of Stuttgart, Germany) presented compelling evidence to show that we can now begin to address this causality dilemma. He outlined how his laboratory, like many others, is exploiting DNA-targeting molecular machines, such as zinc fingers, TALEN and Crispr-Cas9, fused with epigenetic modifiers (for example, histone methyl-tranferases and de-acetylases). The goal is to change the epigenetic information at specific regulatory loci, such as enhancers and promoters, in order to shut down or activate single genes. By fusing *de novo* DNA methyl-tranferases (DNMT3) to small zinc-finger effectors, Jurkowski and colleagues were able to target *EPCAM* and *VEGFA*, two key genes involved in cancer development. More importantly, they demonstrated that adding the DNMT3 co-activator could accelerate the reaction leading to a stronger repression. Interestingly, DNA methylation was found to be highly directional and to reflect the topology of DNA-zinc finger interactions. It was also noted that the number of methylated nucleotides was consistently higher than expected. This phenomenon is attributed to the cooperative binding of individual zinc finger-DNMT3 fusion proteins and can be exploited to increase the desired silencing effect.

DNA de-methylation was also the weapon of choice for James Angstman (Massachusetts General Hospital, USA). In Angstman’s work, the aim was to test how many CpG sites must be de-methylated in order to re-activate gene expression. The data derived from studying the β-globin promoter suggested that not all CpG sites are equivalent and introduced the idea of insulator CpGs, as opposed to regulatory CpGs. Eric Miska (Gurdon Institute at the University of Cambridge and the Wellcome Trust Sanger Institute, UK) reminded the audience that the border between epigenetics and genetics is far more labile than expected. Using *Caenorhabditis elegans*, an organism that lacks DNA methylation altogether, Miska’s group demonstrated how trans-generational gene silencing is mediated by a non-coding RNA mechanism (piwi-RNA). Miska suggested that piwi-RNAs might be seen as the ‘guardian of the epigenome’, with an involvement in maintaining retro-transposon silencing. Use of a different worm that has DNA methylation abilities (*Trichinella spiralis*) hinted at the possibility of RNA-directed DNA methylation, a fascinating hypothesis for mammalian genomics enthusiasts. Finally, Donna Bond (University of Cambridge, UK) presented exciting data from the plant kingdom. Using modified viruses, Bond and colleagues were able to silence (via DNA methylation) single genes epigenetically. The virus itself was not transmitted across generations, but its silencing effects were carried out in the pollen of infected plants and passed on to the new generation.

These results are impressive and demonstrate how epigenetic editing can be effectively used to regulate the expression of single genes reversibly. A problem that has not yet been overcome involves specificity: the zinc finger and CrispR-Cas9 strategies have not yet reached single locus specificity, but they still offer a major advancement compared with the use of genome-wide epigenetic drugs such as 5-azacytidine.

## Histone marks and more

The other face of epigenetic editing deals with another crucial component of epigenetic information: histone modification. Since the seminal work of Allis and colleagues that formalized the theory of the histone code, various laboratories have tried to draw a direct link between covalent tail modifications and transcription. David Cano Rodriguez (University Medical Center Groningen, The Netherlands) gave the audience two clear examples of how we might use histone modifications to ‘wake the sleeping beauties’ (tumor suppressors) and ‘silence the screaming’ (oncogenes). Zinc fingers are extremely promising for this purpose as their chemistry is compatible with *in vivo* usage. They also present several advantages as tools for epigenetic editing when compared to small interfering RNA (siRNA), cDNA or artificial transcription factors. For example, epigenetic editing using zinc fingers offers sustained and inheritable gene suppression without the integration of foreign DNA or siRNA. On the other hand, gene activation is regulated by the endogenous environment and automatically induces the correct gene isoform rather than leading to the non-physiological expression of a pre-determined isoform.

Using zinc fingers fused with the catalytic subunit of the PRC2 repressor complex, Rodriguez and colleagues were able to methylate histone 3 specifically at the Neu/Her2 gene. Her2 expression directly leads to drug resistance in breast cancer and can only be targeted using expensive monoclonal antibodies. The suppression of the Her2 gene was functional and led to a significant reduction in cell proliferation. The transcriptional status of Her2 was also manipulated in the opposite direction, using a transcriptional activator to demonstrate the specificity of zinc-finger targeting. Importantly, whereas activation resulted in *de novo* deposition of some histone marks, H3K9me3 induction was linked to a strong reduction of H3 acetylation and H3 lysine 4 tri-methylation. These data once again highlight the complex relationship between epigenetic information and transcription and recommend caution when discussing the role of epigenetic modifications in isolation.

## Going the distance

A common thread among all of the work described at this meeting was that all of the epigenetic editing was conducted at promoter elements. This approach was adopted to simplify interpretation of the results and to draw a straight line between regulatory regions and the regulated genes. This use of strategy, however, may also explain why the observed changes in transcription were often very subdued but very significant. It was proposed that simultaneous targeting of promoters and associated distal elements, such as enhancers, could dramatically improve our ability to reprogram the epigenetic barcode of cells. In the future, such strategies could lead to efficient differentiation protocols for induced stem cells. Equally important, we can envision a future in which gene manipulation will not necessarily include only genetically engineered intervention but could include a ‘softer’ and more versatile toolkit.

Clinical trials using zinc-finger epigenetic editing are now in phase II for HIV/AIDS and Alzheimer's disease. Studies such as those presented at this meeting let us hope that more and more oncogenes or tumor suppressors will be targeted effectively in the near future of epigenomic medicine.

## Competing interests

The author declares that he have no competing interests.

